# Notes and News

**Published:** 1889-02-16

**Authors:** 


### Notes and News.

Collected and Communicated.

The Rev. N. Bromley has been appointed to the new office of warden of King's College Hospital. This post seems to include the duties of chaplain, performed for many years by Mr. Bromley, and also those of secretary, lately performed by Mr. Moss Macdonald.

The Christmas treat at the Children's Hospital, Coventry, was delayed by the scarlet fever epidemic, and only took place last week. There was the usual tree, games, songs, and, above all, a good tea. Dr. Miller, Miss Easton, and many guests did their best to give the poor little ones a pleasant evening.

Mr. John Norbury presided last week at the annual meeting of the City of London Truss Society. The report read stated that over nine thousand patients were relieved last year, and that the work of the society increases annually. Mr. Kingdon was chosen vice-president, and Mr. Langton and Mr. McCready were appointed surgeons.

We have heard of dislocation of the jaw-bone from yawning, but a far more awful warning to lazy folk comes from Cambridge, U.S. Mr. Lackey of that town, it is said, has ruptured some of the ligaments of the vertebræ by indulging in an extra big yawn, and his condition is considered very critical. In future, however tired we may feel, we had better remember that yawning is not only impolitic, but also dangerous.

A severe outbreak of scarlet fever has occurred in Macclesfield, and the outbreak is primarily traceable to the milk supply, as in the case of the North London outbreak in 1885. It was resolved by the local authorities to ask the Local Government Board to send down an inspector and call in a veterinary surgeon to aid the local medical officer in the investigation.

A series of half-a-dozen interesting articles on the Oldham Infirmary have lately appeared in the *Oldham Evening Express*. The reporter did his work thoroughly, he routed up the matron, Miss Thomson, and the house surgeon, and paid them numerous visits at different times, thus managing to get a very fair idea of the day's work within the wards. The articles ought to rouse the interest of the Oldham people.

Mr. Pritchard presided at the annual meeting of the governors of the Royal Free Hospital on February 7th. The income for last year was £12,327, the expenditure £10,336. The number of in-patients treated was 1,823, and out-patients 21,676. We congratulate the hospital on the improvement in the report, which is well made out. It was announced that Lord Dufferin had consented to become president, probably owing to the interest he takes in the lady medical students who walk this hospital. It was agreed that at the next meeting plans should be considered for the reconstruction of the front portion of the hospital in such a manner as to give increased room for a children's ward, medical offices, etc. Votes of thanks to officers and chairman concluded the proceedings.

The annual meeting of governors and subscribers was held at Durham County Hospital on February 5th. The report for the past year shows that the income has been £2,564 11s. 7½d., and the expenditure £2,032 15s., leaving a balance in hand of the sum of £531 16s. 7½d. The hospital contains 70 beds, and of these an average number of 34 has been occupied each day of the past year. Miss Haythorne, the matron, works well for the institution.

The Convalescent Home at Dover, which has been conducted by Miss Rusher for upwards of thirty years, has been made over in trust to Messrs. J. E. Nichols, G. Vaughan, and W. H. Chinn, for the Manchester Unity of Oddfellows, the South London District of which society will appoint a committee of management. The home has accommodation for eighty convalescents, and, with an endowment fund of £750, represents a gift of more than £3,000.

The quarterly pamphlet of the Edinburgh Medical Missionary Society tells of work which stretches to every quarter of the globe. Of three students who have lately finished their studies, two have been appointed to Manchuria and one to Madagascar. Letters are published in this pamphlet which have been received from members of the society working at Nazareth, Agra, Paris, Scutari, Persia, Travancore, Africa, Paris, and China. In the last country some native gentlemen are building a Mission Hospital for Dr. Shrubshall.

The annual meeting of the Children's Convalescent Home subscribers at Yarmouth was recently held. The Home is in connexion with the Jenny Lind Infirmary, and has sheltered over two hundred small convalescents during the year. This was a large increase on last year, but donations had come in unexpectedly (including an anonymous £100), and the year's work had been one of perfect content and harmony. The housekeeping must be excellently done, for the expenses under that heading only reach £221, though there are five adults in the Home, as well as the patients.

Another microbe caught and labelled! Even as Parisian detectives are unequalled at discovering criminals, so Parisian doctors are unequalled at discovering microbes. There must be a national gift for following up clues and tracing effects to causes in the French character surely, and this is why even the minutest microbe is dragged forth from its infinitesimal lair and made evident before all people. Dr. Roux and Dr. Versin state that the diphtheria microbe is about the same length as that of tuberculosis, but thicker and rounder at the extremities. It is greyish-white in colour, and flourishes in veal broth. This broth, administered to an animal, causes it to die with all the symptoms of diphtheritic poisoning similar to those seen in children. Though the microbe has been found and isolated, the discovery is worth little if it does not lead to knowledge of a cure for diphtheria.

Here is a delightful story anent bone-setting, which will amuse all medical practitioners: "A country lad in the north of Scotland got his leg hurt at one of the local factories. His mother, who had great faith in a neighbouring bone-setter, wanted the lad to go to him. Accompanied by his anxious parent, he was, after a rather painful journey, taken to the town where the bone-setter resided. The leg was duly examined, and it was found necessary to haul it very severely, in order, as the bone-setter said, 'to get the bone in.' The lad was liberal with his screams while this was going on, but eventually the bone was 'got in,' and he was told to go home, and in a few days he would be all right and fit for his work. 'Didn't Danny do the thing well?' said the joyous mother. 'Yes, he did, mither," answered the lad, 'but *I wasna sic a fool as to gie him the sair leg!*'"

The following vacancies are announced this week, the amount of salary in each case being given after the name of the institution :—

**Resident Medical Officers.**

Colchester Asylum for Idiots. Medical Attendant.—£100, board, etc.
Birmingham Asylum. Clinical Assistant.—Board, etc.
South-Eastern Fever Hospital. Clinical Assistant.—Board, etc.
Tunbridge Wells Hospital. House Surgeon and Secretary.—£100, board, etc.

**Non-resident Medical Officers.**

Bristol Provident Medical Institute. Medical Officer.
Owens College, Manchester. Lecturer on Skin Diseases.
St. Pancras Northern Dispensary. Honorary Physician.
Salop Foresters' Medical Association. Surgeon.—£160.
Southern and Maternity Hospitals, Manchester. Surgeon.
Victoria Hospital for Children, Chelsea. Two Physicians for Out-patients.

---

At a meeting of the Board of Management of Salford Royal Hospital, held on January 30th, Mr. George Southam presiding, a letter was read announcing that the late Mr. John Lowcock, of Broughton Park, had bequeathed a legacy of £500 (clear of duty) for the use of the institution. It was proposed to name two cots in the children's ward the "Whitworth" and the "Worral-Walker," in commemoration of legacies lately left to the hospital of £1,000 and £1,396 respectively. The elective committee appointed A. Jasper Anderson, M.A., M.B. Oxon., to the post of honorary medical officer to the Pendleton branch, in place of Mr. A. T. Winterbottom (resigned).

---

At the last meeting of the Bridport Dispensary Committee the secretary spoke very strongly about the medical clubs of the town. He said there were a large number of clubs in the town, legitimate and illegitimate. Amongst the latter he alluded to those in which 1d. or ½d. a-week was paid by families for medical attendance, and strongly deprecated the practice adopted by those who, if not personally, by their agents, had been seeking those families, and seeking to develop this state of things. He thought that was a very strong reason why the numbers of the dispensary had decreased. He would rather give advice free to those who were desirous of it than lower the status of the profession in that way. We advise Mr. Evans to work the dispensary on the provident system; this is by far the best cure for such evils.

---

Perth City and County Infirmary issues a report for 1888, in which it is stated that the total number of dispensary patients treated was 1,034, giving an increase of 214. The district surgeon visited 294. The number of cases admitted to the out-patient room for small accidents, etc., was 253. Forty-five fever cases were brought to the house desultorily throughout the year—scarlatina being most prominent—and the fever wards had therefore always been more or less in use. Accidents, of which a large proportion came from the railway works at Glenfarg, have been unusually numerous, many of them of a very serious nature, some proving fatal almost immediately after admission. The work of the wards has been unusually heavy, the number of patients treated there during the year being 693. Those who attended at the dispensary for advice and medicines received 2,413 consultations, while 610 visits were made by Dr. Gowans to the sick poor at their own homes. These latter patients also received 5,634 visits from the district nurse, who dressed their wounds, ministered to their wants, supplied them with medicine and nourishing food, attended to the order and cleanliness of the sick room, and otherwise carried out the instructions of the district surgeon. The total income from all sources was £2,451 11s. 4½d., showing an increase of £31 19s. 3½d. The expenditure was £2,794 6s. 4d., which gives an increase of £87 8s., while the deficiency for the year was £297 14s. 11½d.

Alderman Sir Robert N. Fowler, M.P., will preside at the sixty-eighth annual court of governors of the Seamen's Hospital Society (late Dreadnought), to be held at Cannon Street Hotel, on Friday, the 22nd inst., when the presence of all friends of the institution, including ladies, is particularly requested. A special court of governors only will be held before the general court.

---

They are enthusiastic in the study of medicine in Scotland. Dr. M'Call Anderson has at present under his care a boy suffering from *xeroderma pigmentosa*, a very rare complaint. In fact, this case is the only one at present known in Scotland, and the interest in it is so great that Dr. Herman Lawrence, an Australian specialist in skin diseases, who is at present in this country, got Dr. Anderson's permission to take the child to Edinburgh, to be shown in Dr. Allan Jamieson's class, where no fewer than two hundred medicals, graduates as well as students, assembled to see it, and to hear the demonstration on its phenomenal points.

---

The great interest shown by the Empress Frederick in all hospitals seems to have aroused the Queen's enthusiasm in this direction also. Not only has her Majesty personally inspected the North Sea Hospital ship since she went to Osborne, but on February 7th her Majesty, accompanied by the German Empress and her daughters, visited the Isle of Wight Infirmary at Ryde. The Royal party drove over from Osborne, and were received by the committee of the charity, the matron, and the medical staff. At her Majesty's request the arrangements for the visit were as simple as possible. The Royal party went over the new wing just completed, and it was then declared open. They then passed through the wards, the German Empress taking great interest in the patients, and addressing a few kind words to each. The Queen accepted a bouquet from the matron and nurses, and expressed her gratification and pleasure at learning that such a well-managed hospital received patients from all parts of the Isle of Wight. After inscribing their names in the visitors' book the Royal party drove through the town, and afterwards returned by road to Osborne.

---

At the annual meeting of Sidmouth Cottage Hospital subscribers on January 28th, Mr. G. Scott had the courage to advocate the plain system of keeping the accounts. Mr. Scott said that last year when he received the annual report, he was surprised to see a deficit of, in round figures, £20 on the year's working, but on looking over the report and balance-sheet he found that a sum of money had been carried to the deposit account instead of being used for paying the bills, and he found if this money had not been placed to the deposit account they would have had a balance in hand instead of a deficit. He thought the management had made a mistake in doing this, as it put their endeavours in a wrong position. That they had done their best he was assured, and the hospital was doing a good work, but he thought they were disparaging their establishment in this matter, as they had this year to clear off the deficit shown, and pay their current expenses as well. The speaker said he spoke from experience, and the plan he suggested he had known to prosper on many occasions. The position of the institution at the present time reflected the greatest credit on the management, and in making the remarks he did it was for the benefit of the hospital, which he was sure they all had at heart. The late action with regard to St. John's Hospital ought to frighten those who desire to set up their own system of accounts. We quite agree with Mr. Scott that when a hospital has a balance, it should acknowledge it and be proud. It is not well to be always crying "Wolf!"

